# Influenza-Like Illnesses in Senegal: Not Only Focus on Influenza Viruses

**DOI:** 10.1371/journal.pone.0093227

**Published:** 2014-03-27

**Authors:** Ndongo Dia, Fatoumata Diene Sarr, Diamilatou Thiam, Tening Faye Sarr, Emmanuelle Espié, Ibrahim OmarBa, Malang Coly, Mbayame Niang, Vincent Richard, for the 4S Network Group

**Affiliations:** 1 Virological Unit, Pasteur Institute in Dakar, Dakar, Senegal; 2 Epidemiology Unit, Pasteur Institute in Dakar, Dakar, Senegal; 3 Ministry of Health, Dakar, Sénégal; 4 World Health Organization, Dakar, Sénégal; Fondazione IRCCS Policlinico San Matteo, Italy

## Abstract

Influenza surveillance in African countries was initially restricted to the identification of circulating strains. In Senegal, the network has recently been enhanced (i) to include epidemiological data from Dakar and other regions and (ii) to extend virological surveillance to other respiratory viruses. Epidemiological data from the sentinel sites is transmitted daily by mobile phone. The data include those for other febrile syndromes similar to influenza-like illnesses (ILI), corresponding to integrated approach. Also, clinical samples are randomly selected and analyzed for influenza and other respiratory viruses. There were 101,640 declared visits to the 11 sentinel sites between week 11-2012 and week 35-2013; 22% of the visits were for fever syndromes and 23% of the cases of fever syndrome were ILI. Influenza viruses were the second most frequent cause of ILI (20%), after adenoviruses (21%) and before rhinoviruses (18%) and enteroviruses (15%). Co-circulation and co-infection were frequent and were responsible for ILI peaks. The first months of implementation of the enhanced surveillance system confirmed that viruses other the influenza make large contributions to influenza-like illnesses. It is therefore important to consider these etiologies in the development of strategies to reduce respiratory infections. More informative tools and research studies are required to assess the burden of respiratory infections in developing countries.

## Background

The International Health Regulations of 2005 advocate enhanced surveillance of events that may constitute a “public health emergency of international concern”. The threats of novel agents of respiratory infection (particularly SRAS in 2003, and a new coronavirus in 2012) and the emergence of influenza A(H1N1)pdm2009 virus have also focused attention on influenza surveillance capabilities worldwide and specifically in developing countries [1]. In Senegal, the National Influenza Center located in the Pasteur Institute of Dakar (IPD) was established in 1974 and has been part of the Global Influenza Surveillance Network (GISN) coordinated by the World Health Organization (WHO) since 1996 [2]. The WHO GISN provides virological information used in the process of selecting strains for the production of influenza vaccines [3]. Indeed, until 2011, the major aim of the Senegalese influenza surveillance had traditionally been to identify the predominant circulating strains in the community, and specifically in Dakar [2].

Because of the high incidence of malaria and the difficulty of distinguishing influenza from other febrile diseases, the burden of influenza in Africa was long believed to be negligible [4]. However, the WHO GISN has documented the circulation of influenza viruses in some African countries [5], [6], although the available data is insufficient for public health decisions to be made [5]. Indeed, the presence of influenza in Africa has been confirmed, but the impact of the disease has not been established [7]. Data describing the seasonality and epidemiology of influenza in tropical areas are limited [4], [5], [8]. Surveillance is required for non-specific indicators, such as visits to health-care centers for influenza-like illness or hospitalization for pneumonia, to provide an indication of the total disease burden. Other respiratory viruses need to be investigated.

The Senegalese influenza surveillance has therefore been enhanced to cover syndromic indicators and the identification of other respiratory viruses. We describe the challenges and steps involved in improving the syndromic sentinel surveillance network in Senegal (4S network), based on nearly real-time principles.

## Methods

The 4S network uses health service-based indicators, and mostly focuses on fever syndromes and diarrhea.

### Management team

The first step in creating the sentinel fever network was to identify the appropriate stakeholders. A steering committee was set up by the Ministry of Health (MoH), the Pasteur Institute in Dakar (IPD) and the WHO local office, and consisted of epidemiologists, virologists and MoH public health managers in charge of the epidemiological surveillance service. Next, immediate external stakeholders, including the regional health department and district managers, were brought into the discussion to identify potential sentinel centers.

### Sentinel primary health care centers

**Figure 1:**
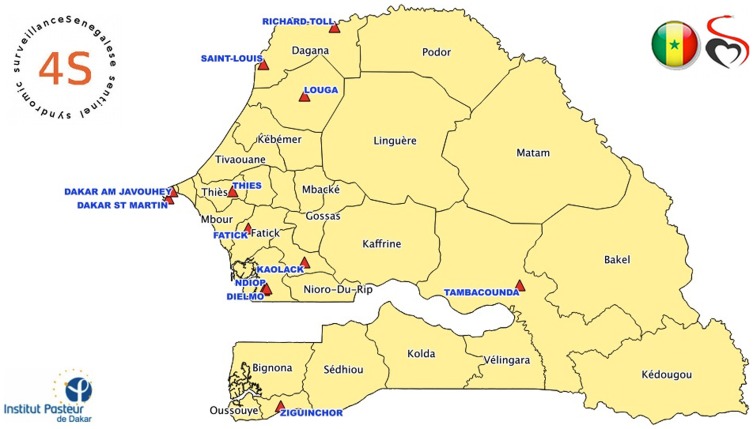
Location of the sentinel sites in Senegal 2013.

As different parts of Senegal have different climate patterns, sentinel centers were established at geographically representative sites so as to provide information about the trends in each of these climate areas. Each sentinel center was selected according to several criteria, including the number of health care workers (at last two per center), care level (activities, number of people that visit the center, equipment), communication facilities (mobile phone network availability) and motivation (voluntary participation) for the surveillance activity. Sentinel general practitioners, recruited on a voluntary and unpaid basis, are the backbone of this system. The network has been expanded from three ILI sentinel sites, all in Dakar, in 2011 to 11 sentinel sites in 2013 (figure 1).

### Data Collection

Surveillance was based on data collected by sentinel general practitioners (SGP). Each participating SGP reported the number of cases that met the criteria, including the total number of patient visits on each reporting day. SGPs were expected to communicate encrypted data by cellular telephone (encrypted SMS) at least once a day, from Monday to Friday, because of health care center closures resulting from routine weekday–weekend schedules. If data from a sentinel center was not received by 10:00 a.m., the IPD staff member contacted the sentinel center to obtain the missing data.

### Case definition

The sentinel surveillance system in Senegal was based on clinical pre-diagnostic data using standard WHO case definitions to ensure comparability. Fever (the inclusion criterion was an axillary temperature of more than 37.5°C) was the first symptom targeted. Three diseases associated with fever were selected for surveillance: confirmed malaria cases (the inclusion criteria were was fever and a positive result in a rapid diagnostic test); influenza-like illness (defined as fever with cough or fever with sore throat); and arbovirus infection (the inclusion criteria were fever without respiratory symptoms and at least two of the following symptoms: headache, arthralgia, myalgia-like backache, skin rash, retro-orbital pain and hemorrhagic manifestations). Diarrheal disease was defined as three or more abnormally loose stools during the previous 24 hours.

Data collection on a daily basis began in March 2012 and continues today. To improve real-time surveillance, we are currently increasing the data entry and data transfer speed, with the aim of collecting most of the data within a 24-hour period. The data obtained daily from the SMS were entered into the Access® database.

### Virological surveillance

Samples were collected from consenting ILI patients at each sentinel site, and subjected to multiplex PCR targeting 16 respiratory viruses for virological surveillance. A two-step real-time RT-PCR was performed using the CFX96 Real-time PCR system (Bio-Rad) and Anyplex II RV16 Detection kit (Seegene). This kit allows simultaneous testing for the presence of Influenza A virus, Influenza B virus, Human respiratory syncytial virus A, Human respiratory syncytial virus B, Human adenovirus, Human metapneumovirus, Human coronavirus 229E, Human coronavirus NL63, Human coronavirus OC43, Human parainfluenza virus -1, -2, -3 and -4, Human rhinovirus A/B/C, Human enterovirus and Human bocavirus [9].

### Data analysis

Data were available for analysis shortly after the patient's initial visit. A medical epidemiologist reviewed the output from all 11 primary health centers on a daily basis, and noted any changes in community-wide incidences of fever. Separate analyses were carried out for each syndrome category of interest, to look for changes in the distribution at each sentinel center.

We estimated the proportion of all fever cases corresponding to each febrile syndrome at each sentinel center; we also estimated the proportion of all fever or diarrhea cases that were primary case encounters.

Thus, various health service-based indicators were monitored daily, including the percentage of fever cases, confirmed malaria cases, influenza-like illness cases, suspected cases of arbovirus infection, and the number of cases with diarrhea and diarrhea with fever.

**Figure 2:**
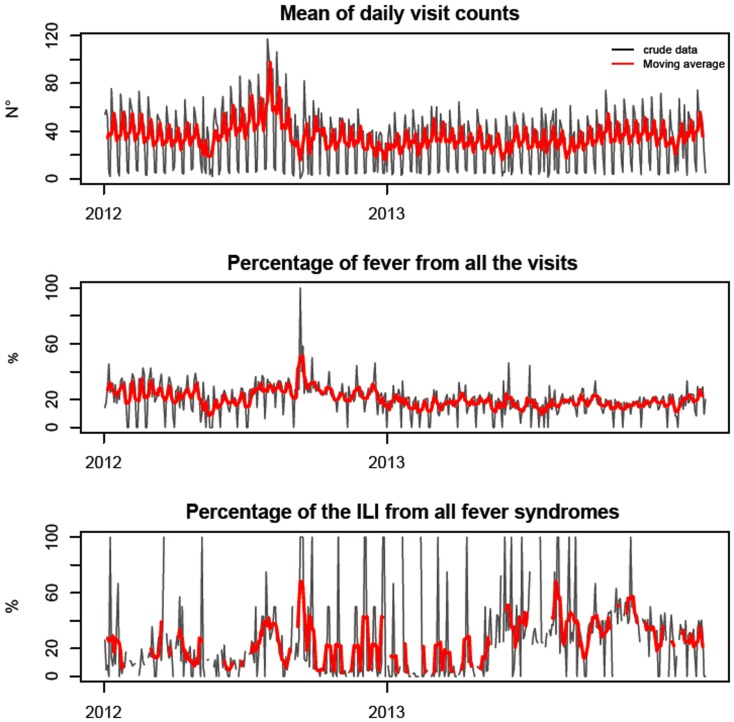
Mean daily visit counts in the sentinel surveillance network in Senegal and daily sentinel surveillance time series plots (%) of total visits for fever, daily visit counts and total ILI for daily total fever syndromes with the moving average (over 10 days – red curve), from Week 11-2012 to Week 35-2013.

Surveillance data analysis was descriptive and straightforward using standard epidemiological techniques. Data are presented in the form of tables and graphs, and included the number of cases relating to each event (figure 2). Data were handled using statistical programs, which analyzed the daily and weekly values of various indicators, allowing a baseline pattern to be established for each syndrome in Senegal. Weekly reports were prepared and transmitted by MoH to regional and district public health staff, the stakeholders of the sentinel sites and the national and international partners of the MoH.

Currently, fluctuations in values at a sentinel center can be monitored on a daily basis, and increases can be detected through data analysis. Increases are reported immediately by telephone to the MoH, to monitor events and to decide on whether to initiate an outbreak investigation.

### Ability to detect outbreaks

The sentinel surveillance system was able to document peaks in the incidence of febrile syndromes, and identify which of arbovirus, influenza and malaria was the most probable cause. If baseline levels were not available, the identification of aberrations was empirical and based solely on detecting peaks of incidence from the daily monitoring of fluctuations. Each increase was reported immediately by phone to regional health officials and public health officials at the MoH. These district managers assisted with the interpretation and follow-up of aberrations in their respective regions.

Further investigations were carried out if a change was detected. The first step in any outbreak investigation is to confirm the signals. Actually, the use of text message codified to identify affected patients may result some mistakes with inclusion of patients whose main medical complaints would be unrelated to the syndromes under surveillance.

### Ethical clearance

The principles of the 4S network were approved by the Ministry of Health in its guidelines for influenza surveillance policy, finalized with the support of Pasteur Institute in Dakar and the Strengthening Influenza Sentinel Surveillance in Africa (SISA) project funded by the WHO [10]. The protocol and oral consent were determined as routine surveillance activity, and therefore non-research by the Senegalese National Ethics committee and the steering committee for 4S network, an entity representing MoH, IPD, WHO and Clinicians in compliance with all applicable National regulations governing the protection of human subjects. Data were collected in an objective of surveillance and are anonymous. The information provided to participants was an informal description of the study. Respiratory specimens were collected, only after informed consent was granted, verbally, to local health care workers by the patients or parents in the case of minors. Oral consent was documented in the patient form with two questions about received information and about oral consent. Patients could refuse to participate, no specimen will be taken. For the surveillance activities, written consent is judged not necessary by the Senegalese national ethics committee, which has also previously approved the work of the National Influenza Center [11]. Collections of non-sensitive data or an observation from normal care in which participants remain anonymous do not require ethics committee review. The patients included in this study were of all ages and consulted the sentinel sites due to influenza-like symptoms; the patients, or parents in the case of minors, accept the tests for respiratory viruses largely because they are free and safe.

## Results

### Description of the epidemiological indicators

From week 11 in 2012 to week 35 in 2013, data was collected on a daily basis such that 101,640 visits were included in the study (Table1). The distribution by age group was 16,025 (15.8%) less than 1-year olds, 27,937 (27.5%) 1-4yr olds, 17,209 (16.9%) 5-14yr olds, 12,093 (11.9%) for 15-24yr olds and 28,373 (27.9) over 24 yr olds. The age distribution at the various sentinel centers, is indicated in Table 2. In total, 22,662 cases (22.3%) of fever were reported with the fewest (9.9%) in Louga and the most (32.3%) in Dakar Centre.

**Table 1:** Distribution of the visits and syndromes counts and percentage, by sentinel sites, from opening date to week 35-2013 included.

	Opening Date	Visits		Febril syndromes		Suspected Malaria		Confirmed Malaria		Suspected Arbovirosis		Influenza-like illnesses		Diarrheas		Febril Diarrheas	
DISTRICTS		N	(%)*	N	(%)**	N	(%)***	N	(%)^#^	N	(%)***	N	(%)***	N	(%)**	N	(%)***
DAKAR CENTRE	31 May 2012	19,237	(18.9)	6,205	(32.3)	1,428	(23.0)	118	(8.3)	483	(7.8)	642	(10.3)	1,101	(5.7)	616	(9.9)
DAKAR SUD	30 May 2012	46,089	(45.3)	10,880	(23.6)	5,249	(48.2)	1,217	(23.2)	21	(0.2)	2,554	(23.5)	2,001	(4.3)	187	(1.7)
FATICK	15 May 2013	1,064	(1.0)	157	(14.8)	34	(21.7)	6	(17.6)	3	(1.9)	54	(34.4)	57	(5.4)	12	(7.6)
KAOLACK	2 July 2013	828	(0.8)	145	(17.5)	54	(37.2)	17	(31.5)	5	(3.4)	26	(17.9)	95	(11.5)	28	(19.3)
LOUGA	5 June 2013	3,540	(3.5)	349	(9.9)	144	(41.3)	8	(5.6)	2	(0.6)	158	(45.3)	278	(7.9)	37	(10.6)
RICHARDTOLL	1 June 2012	5,835	(5.7)	818	(14.0)	610	(74.6)	15	(2.5)	1	(0.1)	68	(8.3)	674	(11.6)	116	(14.2)
SAINT LOUIS	8 April 2013	7,608	(7.5)	1,721	(22.6)	4	(0.2)	0	(0.0)	171	(9.9)	700	(40.7)	556	(7.3)	212	(12.3)
SOKONE	13 March 2012	5,807	(5.7)	795	(13.7)	786	(98.9)	74	(9.4)	8	(1.0)	337	(42.4)	247	(4.3)	25	(3.1)
TAMBACOUNDA	9 April 2013	3,625	(3.6)	438	(12.1)	305	(69.6)	114	(37.4)	5	(1.1)	100	(22.8)	237	(6.5)	34	(7.8)
THIES	27 Febr 2013	2,475	(2.4)	460	(18.6)	202	(43.9)	41	(20.3)	0	(0.0)	75	(16.3)	139	(5.6)	42	(9.1)
ZIGUINCHOR	17 June 2013	5,532	(5.4)	694	(12.5)	203	(29.3)	20	(9.9)	71	(10.2)	443	(63.8)	192	(3.5)	63	(9.1)
**TOTAL**		**101,640**	**(100.0)**	**22,662**	**(22.3)**	**9,019**	**(39.8)**	**1,630**	**(18.1)**	**770**	**(3.4)**	**5,157**	**(22.8)**	**5,577**	**(5.5)**	**1,372**	**(6.1)**

* number of visit by sentinel site reported to the number of all the visits.

** number of syndromes reported to the number of visits by sentinel site.

*** number of syndromes reported to the number of febril syndromes by sentinel site.

# number of confirmed malaria cases reported to the number of the suspected malaria cases by sentinel site.

**Table 2:** Distribution of the visits by sentinel site and by age groups from opening date to week 35-2013, Senegal.

	Visits from 4S network										
	Total	Age group									
		<1 year		1 to 4 years		5 to 14 years		15 to 24 years		25 years and +	
DISTRICTS	N	n	(%)*	n	(%)*	n	(%)*	n	(%)*	n	(%)*
DAKAR CENTRE	19,237	3,238	(16.8)	7,075	(36.8)	3,268	(17.0)	1,435	(7.5)	4,220	(21.9)
DAKAR SUD	46,089	8,149	(17.7)	12,119	(26.3)	7,013	(15.2)	4,839	(10.5)	13,969	(30.3)
FATICK	1064	70	(6.6)	182	(17.1)	221	(20.8)	172	(16.2)	419	(39.4)
KAOLACK	828	102	(12.3)	191	(23.1)	152	(18.4)	118	(14.3)	265	(32.0)
LOUGA	3,540	197	(5.6)	609	(17.2)	780	(22.0)	697	(19.7)	1,257	(35.5)
RICHARDTOLL	5,835	668	(11.4)	1,057	(18.1)	1,005	(17.2)	1,207	(20.7)	1,898	(32.5)
SAINT LOUIS	7,608	1,968	(25.9)	2,955	(38.8)	1,311	(17.2)	537	(7.1)	837	(11.0)
SOKONE	5,807	338	(5.8)	1,028	(17.7)	1,269	(21.9)	1,031	(17.8)	2,140	(36.9)
TAMBACOUNDA	3,625	543	(15.0)	803	(22.2)	616	(17.0)	687	(19.0)	976	(26.9)
THIES	2,475	138	(5.6)	408	(16.5)	469	(18.9)	462	(18.7)	997	(40.3)
ZIGUINCHOR	5,532	614	(11.1)	1,510	(27.3)	1,105	(20.0)	908	(16.4)	1,395	(25.2)
**TOTAL**	**101,640**	**16,025**	**(15.8)**	**27,937**	**(27.5)**	**17,209**	**(16.9)**	**12,093**	**(11.9)**	**28,373**	**(27.9)**

* number of visits by age group reported to number of all the visit by sentinel site.

ILI accounted for 22.8% of fever cases (range: 8.3% in Richard-Toll, in the north of the country, to 63% in Ziguinchor, in the south) (Table 1). The distribution of ILI cases by age group was 1,132 (22.0%) among the under 1-year olds, 2,042 (39.6%) among 1-4yr olds, 960 (18.6%) among 5-14yr olds, 319 (6.2%) among 15-24yr olds and 693 (13.4%) among the over 24 yr olds (Table 3).

**Table 3:** Distribution of the influenza-like illnesses by sentinel site and by age groups from opening date to week 35-2013, Senegal.

	Influenza-like Illnesses																
	Total		Age group														
			<1 year			1 to 4 years			5 to 14 years			15 to 24 years			25 years and +		
DISTRICTS	N	(%)*	N	(%)**	(%)#	N	(%)**	(%)#	N	(%)**	(%)#	N	(%)**	(%)#	N	(%)**	(%)#
DAKAR CENTRE	642	(12.4)	206	(32.1)	(6.4)	355	(55.3)	(5.0)	82	(12.8)	(2.5)	4	(0.6)	(0.3)	2	(0.3)	(0.0)
DAKAR SUD	2,554	(49.5)	504	(19.7)	(6.2)	921	(36.1)	(7.6)	436	(17.1)	(6.2)	168	(6.6)	(3.5)	533	(20.9)	(3.8)
FATICK	54	(1.0)	3	(5.6)	(4.3)	20	(37.0)	(11.0)	13	(24.1)	(5.9)	8	(14.8)	(4.7)	10	(18.5)	(2.4)
KAOLACK	26	(0.5)	7	(26.9)	(6.9)	8	(30.8)	(4.2)	6	(23.1)	(3.9)	2	(7.7)	(1.7)	3	(11.5)	(1.1)
LOUGA	158	(3.1)	15	(9.5)	(7.6)	38	(24.1)	(6.2)	43	(27.2)	(5.5)	19	(12.0)	(2.7)	48	(30.4)	(3.8)
RICHARDTOLL	68	(1.3)	8	(11.8)	(1.2)	12	(17.6)	(1.1)	19	(27.9)	(1.9)	10	(14.7)	(0.8)	19	(27.9)	(1.0)
SAINT LOUIS	700	(13.6)	232	(33.1)	(11.8)	320	(45.7)	(10.8)	112	(16.0)	(8.5)	23	(3.3)	(4.3)	13	(1.9)	(1.6)
SOKONE	337	(6.5)	57	(16.9)	(16.9)	130	(38.6)	(12.6)	75	(22.3)	(5.9)	25	(7.4)	(2.4)	19	(5.6)	(0.9)
TAMBACOUNDA	100	(1.9)	35	(35.0)	(6.4)	37	(37.0)	(4.6)	15	(15.0)	(2.4)	3	(3.0)	(0.4)	10	(10.0)	(1.0)
THIES	75	(1.5)	6	(8.0)	(4.3)	25	(33.3)	(6.1)	25	(33.3)	(5.3)	9	(12.0)	(1.9)	10	(13.3)	(1.0)
ZIGUINCHOR	443	(8.6)	59	(13.3)	(9.6)	176	(39.7)	(11.7)	134	(30.2)	(12.1)	48	(10.8)	(5.3)	26	(5.9)	(1.9)
**TOTAL**	**5,157**	**(100.0)**	**1,132**	**(22.0)**	**(7.1)**	**2,042**	**(39.6)**	**(7.3)**	**960**	**(18.6)**	**(5.6)**	**319**	**(6.2)**	**(2.6)**	**693**	**(13.4)**	**(2.4)**

* number of ILI reported to the total number of ILI.

**number of ILI reported to the number of all ILI by sentinel site.

# number of ILI reported to the number of visits by sentinel site et age group.

Dengue-like syndromes accounted for 3.4% of fever cases and suspected malaria cases for 39.8%; 18.1% of suspected malaria cases were confirmed (Table1).

Diarrhea cases accounted for 5.5% of visits and febrile diarrhoeas for 6.1% of febrile syndromes and 24.6% of diarrheas (Table 1).

The epidemiological characteristics of groups with fever-related syndromes, such as those with ILI, identified by the sentinel surveillance system, were investigated graphically. Total daily counts (Figure 2), and daily and weekly counts for each sentinel center (data not shown), were plotted and analyzed. This revealed a peak in October 2012 corresponding to an increase in the number of febrile syndromes and ILI cases.

**Figure 3:**
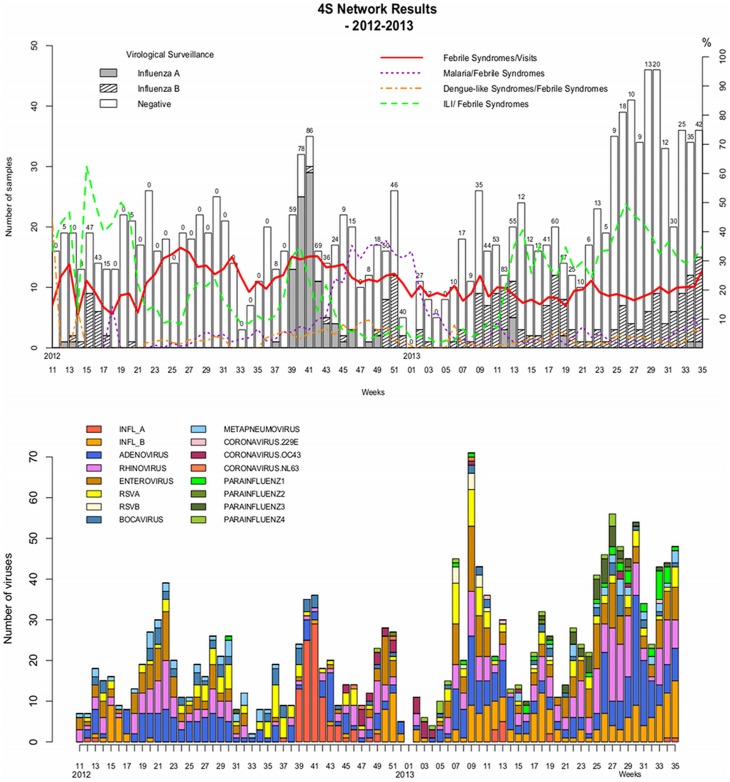
Data from all the sentinel sites by week, 2012–2013.

**Figure 4:**
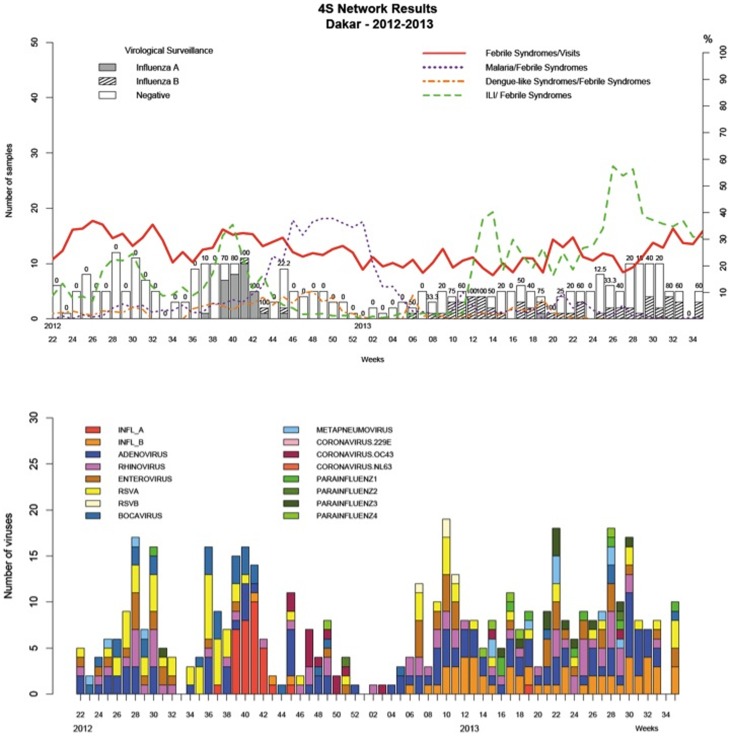
Data from Dakar by week, 2012–2013.

A plot of the incidence of febrile and other syndromes over the time (figures 3 and 4) showed that fever syndromes made up between 10% and 30% of cases and ILI was the dominant cause of fever throughout the study period in most of the country, except during the malaria season from week 43-2012 to week 6-2013.

### Virological surveillance

Specimens (n = 1427) were collected from all the age groups. However, the percentages of sampled ILI patients were statistically different between the age groups with respectively 12% (133/1132) of sampled ILI patients among the under 1-year olds, 32% (651/2042) among 1-4yr olds, 38% (368/960) among 5–14yr olds, 43% (138/319) among 15–24yr olds and 19% (137/693) among the over 24yr olds (p<0.001).

Of the 1427 patients sampled, 389 were negative (27%), and 1678 viruses were detected in the 1038 positive patients (73%): 363 adenoviruses (22%), 317 Influenza viruses (19%), 210 influenza B viruses (13%), 107 influenza A (H3N2) viruses (6%), 311 rhinoviruses (19%), 257 enteroviruses (15%), 158 respiratory syncytial viruses (9%) (143 type A and 15 type B), 99 parainfluenza viruses (6%), 79 bocavirus (5%), 56 human metapneumoviruses (3%), and 38 coronaviruses (2%) (Table 4).

**Table 4:** Virus distribution according with sentinel sites between week 11-2012 and week 35-2013 in Senegal.

DISTRICT	Number of samples		Negative results		Total Viruses		Adenov.		Rhinov.		Influenza A		Influenza B		Enterov.		RSV A		RSV B	
	n	(%)	n	(%)	n	(%)	n	(%)	n	(%)	n	(%)	n	(%)	n	(%)	n	(%)	n	(%)
**DAKAR CENTRE**	254	(18)	42	(17)	358	(21)	77	(22)	50	(14)	21	(6)	44	(12)	39	(11)	53	(15)	4	(1)
**DAKAR SUD**	126	(9)	27	(21)	162	(10)	37	(23)	25	(15)	13	(8)	17	(10)	21	(13)	22	(14)	0	(0)
**FATICK**	37	(3)	8	(22)	43	(3)	4	(9)	10	(23)	0	(0)	7	(16)	4	(9)	4	(9)	0	(0)
**KAOLACK**	19	(1)	3	(16)	27	(2)	6	(22)	6	(22)	1	(4)	2	(7)	4	(15)	1	(4)	0	(0)
**LOUGA**	41	(3)	10	(24)	52	(3)	10	(19)	11	(21)	0	(0)	7	(13)	10	(19)	4	(8)	0	(0)
**RICHARDTOLL**	62	(4)	23	(37)	57	(3)	19	(33)	10	(18)	7	(12)	4	(7)	6	(11)	6	(11)	0	(0)
**SAINT LOUIS**	36	(3)	11	(31)	42	(3)	13	(31)	7	(17)	0	(0)	7	(17)	5	(12)	5	(12)	0	(0)
**SOKONE**	730	(51)	251	(34)	751	(45)	166	(22)	161	(21)	65	(9)	71	(9)	139	(19)	35	(5)	6	(1)
**TAMBACOUNDA**	13	(1)	1	(8)	19	(1)	3	(16)	3	(16)	0	(0)	0	(0)	6	(32)	0	(0)	0	(0)
**THIES**	52	(4)	7	(13)	95	(6)	20	(21)	13	(14)	0	(0)	24	(25)	13	(14)	12	(13)	5	(5)
**ZIGUINCHOR**	57	(4)	6	(11)	72	(4)	8	(11)	15	(21)	0	(0)	27	(38)	10	(14)	1	(1)	0	(0)
**TOTAL**	**1427**	**(100)**	**389**	**(27)**	**1678**	**(100)**	**363**	**(22)**	**311**	**(19)**	**107**	**(6)**	**210**	**(13)**	**257**	**(15)**	**143**	**(9)**	**15**	**(1)**

DISTRICT	Coron. 229E		Coron. OC43		Coron. NL63		ParaInfl. 1		ParaInfl. 2		ParaInfl. 3		ParaInfl. 4		Meta Pneumov.		Bocav.	
	n	(%)	n	(%)	n	(%)	n	(%)	n	(%)	n	(%)	n	(%)	n	(%)	n	(%)
**DAKAR CENTRE**	0	(0)	14	(4)	0	(0)	6	(2)	2	(1)	12	(3)	3	(1)	14	(4)	19	(5)
**DAKAR SUD**	0	(0)	0	(0)	0	(0)	4	(2)	0	(0)	1	(1)	3	(2)	3	(2)	16	(10)
**FATICK**	0	(0)	1	(2)	0	(0)	0	(0)	0	(0)	5	(12)	0	(0)	5	(12)	3	(7)
**KAOLACK**	0	(0)	0	(0)	0	(0)	2	(7)	0	(0)	1	(4)	2	(7)	2	(7)	0	(0)
**LOUGA**	1	(2)	0	(0)	0	(0)	1	(2)	0	(0)	4	(8)	0	(0)	2	(4)	2	(4)
**RICHARDTOLL**	0	(0)	0	(0)	0	(0)	1	(2)	0	(0)	0	(0)	0	(0)	1	(2)	3	(5)
**SAINT LOUIS**	0	(0)	1	(2)	0	(0)	0	(0)	2	(5)	0	(0)	0	(0)	2	(5)	0	(0)
**SOKONE**	2	(0)	15	(2)	4	(1)	14	(2)	0	(0)	8	(1)	8	(1)	24	(3)	33	(4)
**TAMBACOUNDA**	0	(0)	0	(0)	0	(0)	2	(11)	0	(0)	2	(11)	0	(0)	0	(0)	3	(16)
**THIES**	0	(0)	0	(0)	0	(0)	4	(4)	0	(0)	0	(0)	1	(1)	3	(3)	0	(0)
**ZIGUINCHOR**	0	(0)	0	(0)	0	(0)	1	(1)	0	(0)	5	(7)	5	(7)	0	(0)	0	(0)
**TOTAL**	**3**	**(0)**	**31**	**(2)**	**4**	**(0)**	**35**	**(2)**	**4**	**(0)**	**38**	**(2)**	**22**	**(1)**	**56**	**(3)**	**79**	**(5)**

Mono-infections were found in 588 positive patients (57%), dual-infections in 312 (30%) and more than two viruses in 138 (13%) (Table 5).

**Table 5:** Virus co-infections between week 11-2012 and week 35-2013 in Senegal.

	INFL A	INFL_B	RSV A	RSV B	ADENO	RHINO	CORON	PARINF	METAPN	BOCA	ENTERO
INFLUENZAE_A	70	5	1	0	18	9	1	0	2	8	6
INFLUENZAE_B		103	25	7	55	30	3	3	5	3	39
RSV A			50	11	46	28	0	7	4	10	49
RSV B				1	7	5	0	0	0	1	11
ADENOVIRUS					155	92	6	16	12	21	73
RHINOVIRUS						70	7	27	12	21	142
CORONAVIRUS							22	0	0	2	3
PARAINFLUENZAE								42	1	1	28
METAPNEUMOVIRUS									28	5	11
BOCAVIRUS										22	17
ENTEROVIRUS											25
MonoInfection	70	103	50	1	155	70	22	42	28	22	25
Dual infection	27	65	42	1	114	147	9	39	12	35	133
Multi-infection	10	42	51	13	94	94	7	18	16	22	99
**Total**	**107**	**210**	**143**	**15**	**363**	**311**	**38**	**99**	**56**	**79**	**257**

There were 363 positive patients for adenovirus infection, of which 155 displayed mono-infections (26% of all mono-infections), 114 dual-infections (37% of all dual-infections) and 94 more than two viruses (68% of all the multi-infections).

There were 312 positive patients for influenza virus infection, of which 173 displayed mono-infections (29% of all mono-infections), 89 dual-infections (29% of all dual-infections) and 50 more than two viruses (36% of all the multi-infections).

**Figure 5:**
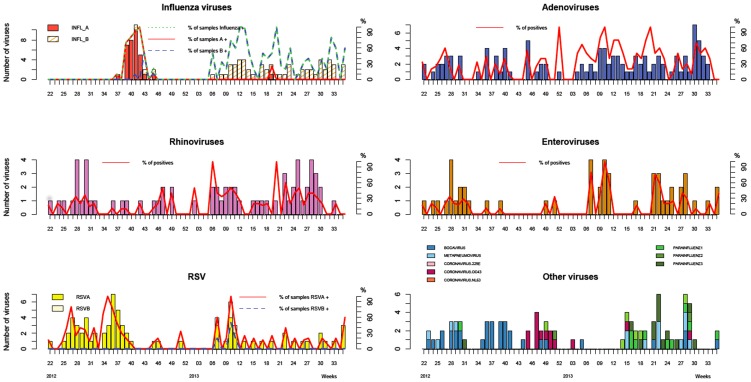
Distribution of different respiratory viruses in Dakar by week, 2012–2013.

There were peaks of ILI incidence in Dakar during the study period (Figure 4). The first was from week 26-2012 to week 31-2012 as was associated with the co-circulation of rhinoviruses, enteroviruses and respiratory syncytial viruses; the second from week 38-2012 to week 42-2012 was associated with a peak in the incidence of Influenza A(H3N2). Between week 10-2013 and week 35-2013, the ILI incidence was high, due persistent co-circulation of influenza B, adenovirus and rhinovirus. This period also included two peaks: from week 11-2013 to week 15-2013 due to enterovirus and RSV; and from week 25-2013 to week 29-2013 due to parainfluenza virus (Figures 4–5).

## Discussion

There is a history of influenza surveillance in Senegal [12] and this surveillance has been more effective than that in many African countries. The aim of the Senegalese influenza surveillance has been to identify the influenza strains predominantly circulating in the community in Dakar [2]. More recently, the Senegalese influenza surveillance system has been further improved with the support of the Pasteur Institute in Dakar.

The improvements include associating a syndromic surveillance to virological surveillance, establishing real time-like surveillance principles for epidemiological data collection as in Madagascar [13], [14], broadening the virological surveillance to other respiratory viruses causing similar symptoms to influenza viruses, and to including sites in geographically diverse areas with different climates. Consequently, the system is no longer limited to identifying the predominant circulating strains. It can also estimate the significance of ILI to health care activity, through syndromic surveillance. Furthermore, other syndromes have been included in the surveillance, and in particular, the network has been extended to cover suspected malaria cases, dengue-like syndromes and diarrhea.

This improved Senegalese surveillance system, now called the 4S network, is based on reporting nonspecific indicators as epidemiological data to the health-care authorities, and on random sampling for laboratory-based surveillance. However, none of the indicators are accurate measures of incidence. Indeed, although the new tools used by the 4S network since 2011 are convenient, the 4S network is still not yet able to evaluate the relative contribution of influenza and other respiratory viruses to disease burden, or child or adult mortality. Consequently, the influenza surveillance in Senegal could be improved by collecting complementary data. In particular, mortality data and more accurate incidence data would help assessments of the impact of influenza and the design of appropriate preventive strategies. Until such data are available, it difficult to justify the widespread use of influenza vaccine or other influenza control measures. Nevertheless, the health burden associated with influenza may be far greater in Africa than in developed countries, due to co-circulating tropical disease, immunosuppression and malnutrition [15]. Thus, the next step of the 4S network will be to collect data corresponding to hospital admissions for acute respiratory infection (ARI) and to ARI mortality. Hospital-based sentinel surveillance can provide substantial insights into the contribution of influenza infection to the global burden of disease [3]. A clear understanding of the epidemiology of severe influenza-associated disease is essential for decisions about vaccine recommendations.

The 4S network will implement population-based sentinel surveillance, with reporting by mobile phone, to evaluate the incidence of the respiratory infections in the community [1]. Mobile phones have been available in Africa since the beginning of the XXI century are now widely used by the population; they may be useful to collect information through text messaging.

Influenza virus was found throughout the period during which the network was being enhanced. Influenza A virus was predominant after the rainy season in 2012 but influenza B virus has been found continually during 2013. Determining the current burden of influenza is difficult because seasonality has seemed to be less evident in Senegal since the pandemic linked to the influenza A(H1N1)pdm2009 virus. These results were not similar to those from 1996 to 2009 [2], but were similar to the results of a survey of influenza epidemiology and seasonal variation in Africa [5]: the authors noted influenza activity during all seasons.

However, influenza viruses seem to be only one of several frequent etiological agents of ILI: we found numerous other respiratory viruses, some in mono-infections and some in co-infections indicating that the circulation of these viruses in the community is substantial. As in Madagascar [16], [17], pathogenic respiratory viruses were present in more than 70% of the samples collected from cases of ILI, and the involvement of non-influenza viruses was substantial: adenoviruses (22%), rhinoviruses (19%) and enteroviruses (15%) were the non-influenza viruses most frequently found. In Madagascar[16], HMPV (25%) and RSV (21%) were the most common viruses after influenza viruses. In Tunisia [18], the most common non-flu pathogen circulating in three seasons causing the lower respiratory tract infections especially in children was RSV (45%) but others viruses were also identifed: adenoviruses (20%) during season 2008–2009 or rhinoviruses (44%) during season 2009–2010. In 2009 in Cameron [19], influenza virus and rhinovirus were the most commonly detected viruses. All these results emphasises the importance of countries being able to test for a suite of respiratory pathogens, not just influenza. However, further investigations are necessary for a better understanding of the annual circulation profile of these different respiratory viruses. Besides, the difference between sampling percentages according with age group shows the difficulty to analyze alone virological data, and highlights the need to link virological and epidemiological data to improve the sentinel surveillance systems.

The results reported here are those for the entire network, but are difficult to interpret because most are from sentinel sites in Dakar, and very few are from sites that opened only recently. As more data is collected and becomes available, it will be possible to document the patterns and characteristics of fever syndromes and ILI, both nationally, and in the various regions.

## Conclusion

The 4S network is the first nationwide real-time-like surveillance system established in Senegal. One of the main strengths of this system is its low cost and ease of implementation. Systems like this for collecting epidemiological data can easily be established in medium or low-resource countries. The laboratory-based surveillance costs are greater and therefore international support for funding is required. Much needs to be done to increase awareness about the health burden due to respiratory virus such that better preventive strategies can be implemented.
