# Correction: Influenza-Like Illnesses in Senegal: Not Only Focus on Influenza Viruses

**DOI:** 10.1371/journal.pone.0101722

**Published:** 2014-06-25

**Authors:** The PLOS ONE Staff

There is an error in the spelling of the sixth author’s name. The correct name is: Ibrahim Omar Ba.
